# An Intelligent Motor Assessment Method Utilizing a Bi-Lateral Virtual-Reality Task for Stroke Rehabilitation on Upper Extremity

**DOI:** 10.1109/JTEHM.2022.3213348

**Published:** 2022-10-20

**Authors:** Chia-Ru Chung, Mu-Chun Su, Si-Huei Lee, Eric Hsiao-Kuang Wu, Li-Hsien Tang, Shih-Ching Yeh

**Affiliations:** Department of Computer Science and Information EngineeringNational Central University 34911 Taoyuan 320 Taiwan; Department of Physical Medicine and RehabilitationTaipei Veterans General HospitalNational Yang-Ming University 34882 Taipei 11221 Taiwan

**Keywords:** Stroke rehabilitation, motor training, virtual reality, machine learning

## Abstract

Virtual reality (VR) has been widely adopted by therapists to provide rich motor training tasks. Time series data of motion trajectory accompanied with the interaction of VR system may contain important clues in regard to the assessment of motor function, however, clinical evaluation scales such as Fugl-Meyer Assessment (FMA), Wolf Motor Function Test (WMFT), and Test D’évaluation Des Membres Supérieurs Des Personnes Âgées (TEMPA) are highly depended in clinic. Further, there is not yet an assessment method that simultaneously consider motion trajectory and clinical evaluation scales. The objective of this study is to establish an evidence-based assessment model by machine-learning method that integrated motion trajectory of a VR task with clinical evaluation scales. In this study, a VR system for upper-limb motor training was proposed for stroke rehabilitation. Clinical trials with 20 stroke patients were performed. A variety of motor indicators that derived via motion trajectory were proposed. The correlations between motor indicators and clinical evaluation scales were examined. Further, motor indicators were integrated with evaluation scales to develop a machine-learning based model that represents an evidence-based motor assessment approach. Clinical evaluation scales, FMA, TEMPA and WMFT, were significantly progressed. A few motor indicators were found significantly correlated with clinical evaluation scales. The accuracy of machine-learning based assessment model was up to 86%. The proposed VR system is validated to be effective in motor rehabilitation. Motor indicators derived from motor trajectory were with potential for clinical motor assessment. Machine learning could be a promising tool to perform automatic assessment. Clinical and Translational Impact Statement—A VR task for motor rehabilitation was exanimated via clinical trials. Integrating motor indices with clinical assessment, a machine-learning model with accuracy of 86% was developed to evaluate motor function.

## I. Introduction

Stroke is a leading cause of death in low- and middle- income countries. Globally, 70% of strokes and 87% of both stroke-related deaths and disability-adjusted life years occur in these developing countries [1]. Stroke causes brain death due to poor blood flow into cells inside the brain [2].

In traditional stroke rehabilitation, relevant nerve tissue is stimulated via continuous motor training to restore as much motor function as possible [3], [4], [5]. However, long-term traditional physical rehabilitation tasks become routine and monotonous, leading to a lack of motivation in patients. To assess motor function, doctors and therapists often use traditional evaluation scales like Fugl-Meyer Assessment (FMA) [6], Wolf Motor Function Test (WMFT) [7], and Test D’évaluation Des Membres Supérieurs Des Personnes Âgées (TEMPA) [8]. However, traditional evaluation scales are subjective and a therapist is required for each assessment. This would become an issue if therapists were limited [9].

The goal of interactive training tasks is to increase motivation and concentration in patients. We could achieve this goal through VR. Virtual reality is a computer-based technology that allows users to interact with simulated environments [10]. Virtual reality is more accessible and affordable in motor rehabilitation, increasing patient motivation to perform repetitive rehabilitation tasks [11]. Therefore, much research has focused on VR-based motor rehabilitation [12], [13], [14]. There is also much research demonstrating that VR could lead to significant improvement in stroke rehabilitation [15], [16], [17], [18]. Furthermore, VR could help patients who are un- willing to perform stroke rehabilitation tasks [19]. Moreover, the patient’s training goals could be adjusted according to their physical conditions [20].

Virtual-reality stroke-rehabilitation systems are also able to collect a huge amount of sensing data (motion trajectory, electromyography) via sensors [21], [22], [23], [24]. In most research, VR stroke-rehabilitation systems are only used for motor training, and patient skeleton and electromyography data are collected by the sensors. However, because therapists mainly score evaluation scales, this research did not utilize the physiological data to evaluate the patient’s condition. Although, some research [25], [26], [27] has analyzed the patient’s physiological data collected from sensors in order to implement motor assessment functions and help patients to understand their rehabilitation progress. However, research has not discussed the correlation between proposed motor indicators and traditional evaluation scales.

Artificial intelligence and machine learning have developed rapidly in recent years and are widely used in data mining, computer vision, natural language processing, speech recognition, etc. In the medical domain, some researchers use the clustering method (K-means) to cluster impairment levels [28]. Some researchers use supervised machine learning algorithms to classify a patient’s status [29], [30]. However, due to a lack of correlation with traditional evaluation scales and clinical references, therapists are unconvinced of its effectiveness and capabilities.

To address the issues mentioned above, we wanted to build an evidence-based motor assessment. Our research designed motor indicators with upper-limb data collected by sensors and analyzed the correlation between motor indicators and evaluation scales. Furthermore, we applied machine learning methods to establish an assessment model based on evidence- based motor indicators.

## II. Related Work

In recent years, many researchers have adopted VR training tasks in stroke rehabilitation. Some research has proved the efficiency of VR rehabilitation by comparing pretest and posttest data. Sheehy et al. [31] verified the feasibility of using home-based VR post stroke via a parallel randomized feasibility trial. Aramaki et al. [32] verified that VR is a viable tool for rehabilitating stroke patients with the Canadian Occupational Performance Measure (COPM) and the Participation Scale. Lee et al. [33] verified game-based VR canoe paddling training as an effective rehabilitation therapy using a modified functional reach test (mFRT) and a manual function test (MFT). Choi and Paik [34] proposed a mobile VR upper- extremity rehabilitation program and verified its effects using FMA-UE, B-stage, and manual muscle testing. Although this research has shown the efficiency of VR rehabilitation tasks, physiological data collected from the sensors was not sed to assess the stroke patient’s status. Therefore, therapists were unable to assess a stroke patient after using VR rehabilitation tasks.

Recently, some research has extracted features from sensor data during rehabilitation tasks to assess stroke patients. Phienphanich et al. [25], Lu et al. [26], Lee et al. [27] and Kashi et al. [35] extracted movement features based on biomechanical metrics, including velocity, jerk, index of curvature, and angles of the joints. These movement features considered range of motion, smoothness, and compensation [27]. However, this research did not consider the correlation with traditional evaluation scales (such as FMA, WMFT, or TEMPA). Lee et al. [36] extracted features based on the linguistic guideline of the FMA. However, many other traditional evaluation scales are used to assess a stroke patient’s impairment level. Considering features from the FMA only might not be enough to assess a stroke patient.

Some research has proposed a classification method according to movement features to classify a patient’s impairment level. Biswas et al. [37] used the regularized Mahalonobis distance-based K-means clustering to classify elementary arm movements to different levels of impairment. Miao et al. [28] presented a DTW-KNN joint algorithm to classify multiple training completion levels. Kashi et al. [35] used the RAndom k-labELsets (RAkEL) algorithm via a random forest as the base classifier to establish a multi-label classification model based on stroke patient data. Each of these studies use movement features to implement clustering. However, their clustering methods did not consider traditional evaluation scales. Evaluation scales could provide a reliable, convincible analysis aspect to doctors, therapists, and patients. Our re- search proposes a classification method based on traditional evaluation scales.

Based on the movement data of VR rehabilitation tasks, some research proposed motor assessment methods to provide therapists and patients with a quantitative result to verify improvements of the stroke patient. Wang et al. [38] proposed a system where the patient can complete treatment during a rehabilitation game. Nevertheless, the quantitative results of this paper’s rehabilitation system were not validated using traditional evaluation scales. Liao et al. [39] proposed scoring functions for the automated assessment of the quality of physical rehabilitation tasks. However, these scoring functions also lacked correlation with traditional evaluation scales.

Our study presents VR stroke-rehabilitation tasks containing a Kinect sensor to collect upper-limb movement data. A bi-lateral VR task, including the ball-throwing and ball-catching with two arms back and forth, is designed to train weight-shifting and eye-hand coordination. We propose an evidence-based analysis approach that contains upper-limb movement features, a multi-classification method, and a machine learning approach. We extracted features from upper-limb movement data collected from Kinect and used statistics analysis to verify the correlation between upper- limb movement features and traditional evaluation scales. For the multi-classification method, we utilized the K-means clustering algorithm [37] to divide patient impairment levels into three categories based on the traditional evaluation scales. In comparison to previous studies, our VR stroke-rehabilitation tasks have higher reliability and interpretable ability.

## III. Method

### A. Virtual Reality System Design

#### 1) System Introduction

**FIGURE 1.**
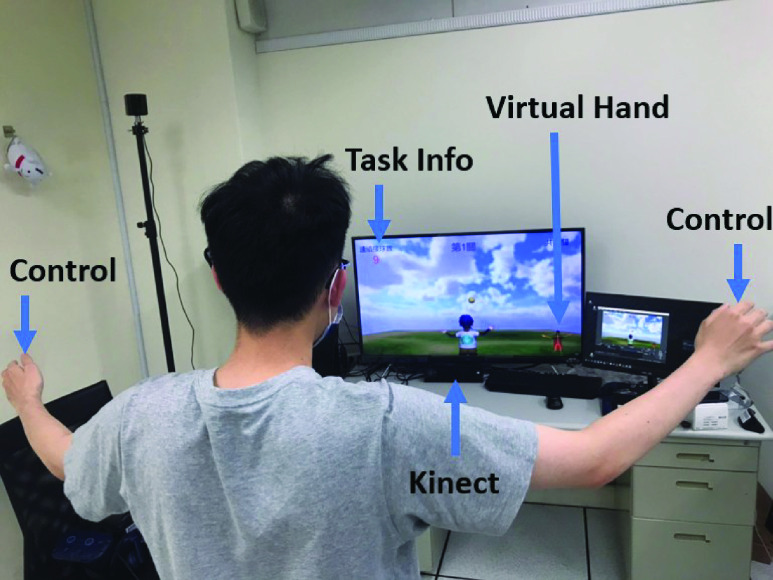
Physical setting of our VR stroke-rehabilitation system which combines Kinect [40], 3D VISION stereo glasses, a 3D display projector, a 3D display card, and other hardware devices. A bi-lateral VR task for upper-limb extension, balance, and hand-eye coordination with the Unity 3D engine were included in this system.

Our VR stroke-rehabilitation system combines Kinect [40], 3D VISION stereo glasses, a 3D display projector, a 3D display card, and other hardware devices. It aims to provide patients with rehabilitation tasks for their upper limbs. It includes a bi-lateral VR task for upper-limb extension, balance, and hand-eye coordination with the Unity 3D engine. The physical setting of our VR stroke-rehabilitation system is shown in Fig. 1.

#### 2) Task Content

**FIGURE 2.**
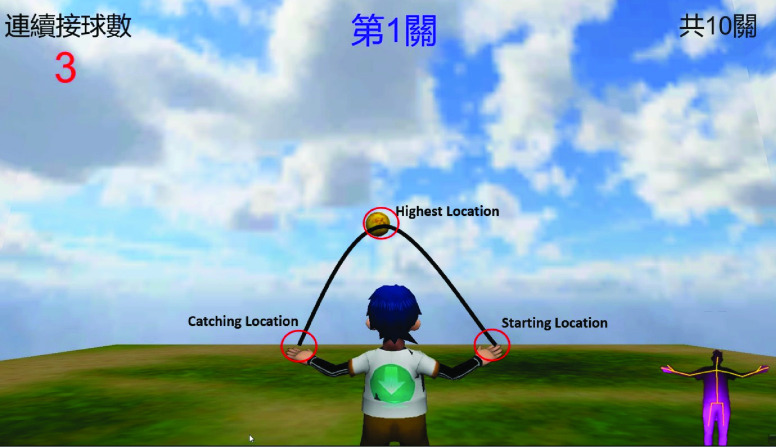
Illustration of bi-lateral VR task. At the beginning of the task, a ball appeared at Starting Location which was at the left side or right side upon the setting. The user had to touch the ball with the hand closer to it, triggering the ball to move along the parabola to the other side, and catch the ball with another hand back and forth.

To train the extension and muscle function of the patient’s arms, the VR task in our system include both throwing and catching a ball with both arms. The participate is asked to stretch both arms to catch the ball continuously, which is moving in a parabola. Kinect was applied to perform motion capture. Before starting the task, therapists set the difficulty of the task that includes the speed of the ball, the range of the flying, and the number of balls to catch. Further description for the difficulty of the task would be illustrated in the coming section. At the beginning of the task, a ball appeared at starting location which was at the left side or right side upon the setting, as shown in Fig. 2. The user had to touch the ball with the hand closer to it, triggering the ball to move along the parabola to the other side, and catch the ball with another hand back and forth. Meanwhile, the user can see his/her current performance including the number of successful ball-catching movements, the number of failed ball-catching movements, and the number of consecutive successful ball-catching movements.

#### 3) Difficulty Design Mechanism

The difficulty of the bi-lateral VR task can be set using the following parameters.

• The horizontal distance of the sphere falling: the distance can be set according to the patient’s range of upper-limb extension. The bi-lateral VR task provides various range options to suit the patient’s upper-limb abilities: 30% to 50%, 30% to 75% and 30% to 100% of the patient’s arm when his/her arm was totally strengthened.
• The number of successful catches: therapists can adjust the number to test how long the patient could continue exercising.
• The speed of the sphere falling: speed is in units of gravitational acceleration ( $G$). Therapists could set this to 1/9G, 2/9G, and 3/9G so that patients could train their reaction time and hand-eye coordination according to their impairment level.

### B. Final Stage

After many discussions with therapists, we determined what therapists want most in regards to patients’ movement quality: reaction time, hand-eye coordination quality, range of upper limb movement, a comparison between the affected arm and the normal arm, and the ball’s speed difference between front and rear at the highest point. Note that the range of upper limb movement for the affected and the normal arm might be different. Evaluating their difference would be critical. The importance of ball speed is the patient’s preparation time and reaction time. When a patient’s arm is injured, his/her muscle would have difficulty controlling which could be evaluated by different ball speeds. In other words, different ball speeds would affect how long patients take to raise their hands and prepare. Assume that after the right hand touches the ball, the left hand must be raised to the target position before landing. It is not an easy task for the patients whose arm is injured. We designed motor indicators in accordance with therapists’ needs, and the schematic diagram is shown in Fig. 2. Features 1 through 6 are related to horizontal and vertical motor ability based on different axes in the upper-limb’s skeleton data. Features 7 through 19 are related to the motor ability of the whole body. The major reason for differentiating the velocity before of the arm when the ball is at the highest location or after was that when the ball is at the highest point, the user should know that the landing point of the ball, and then moves his/her arm. Hence, evaluating the spending time could estimate the patient’s reaction ability.

#### 1) First Time Entering the Catching Range

Record the time when the patient first moves their arm in the catching range

#### 2) Last Time Entering the Catching Range

Record the time when the patient last moves their arm in the catching range.

#### 3) Catching Time

Record the time the patient successfully catches each ball.

#### 4) Aiming Time

Record the time when the patient’s hand moves into the scope of the ball-catching range after the ball arrives at its highest location. A lower value means the patient has a faster reaction time.

#### 5) Amount of Time Between Stabilization and Catching Ball

If a patient receives a higher difference in time than before, this means the patient has a higher degree of control, and the hand tremor is improving. Stabilization indicates the degree of stability of the muscle, which means that whether this movement of the hand trajectory is smooth.

#### 6) Number of Times the Patient Gets in and Out of Catching Range

If the number of times decreases, then the patient’s upper- limb function is recovering.

#### 7) Time Difference Catching the Ball From the Normal Arm to the Affected Arm

Observing the patient’s arm reactions before the ball arrives at its highest location.

#### 8) Ball-Catching Time Cost by Affected Arm

The total time spent touching (catching) the ball with the affected arm during the game. A longer time cost means the patient has a better reaction ability.

#### 9) The Total Length That the Affected Arm Joint Points Moved During Task

Observe the patient’s extension ability of the affected arm.

#### 10) Movement Difference Between the Affected Arm and the Normal Arm

the calculation formula is as shown in

\begin{equation*} \frac {The~ movement~ distance ~by ~normal~ arm}{The~ movement~ distance~ by~ affected ~arm} \tag{1}\end{equation*}

#### 11) Maximum Speed by the Affected Arm Before the Ball Arrives at the Highest Location

the calculation formula is as shown in

\begin{equation*} V_{MAX }=M A X\left ({V_{i}=\frac {\Delta D_{i}}{\Delta t_{i}}}\right)\tag{2}\end{equation*}

where $V_{MAX}$ is the maximum instantaneous speed; $\triangle D_{i}$ is the instantaneous displacement distance; $\triangle t_{i}$ is the instantaneous time difference.

#### 12) Average Speed by Affected Arm Before Ball Arrives at the Highest Location

the calculation formula is as shown in

\begin{equation*} \bar {v}=\frac {1}{N}\left ({\sum \nolimits _{i=1}^{N} V_{i}}\right)\tag{3}\end{equation*}

where $\bar v$ is the average speed; $v_{i}$ is the instantaneous speed; $N$ is the total number of seconds of training.

#### 13) V-Variation by Affected Arm Before Ball Arrives at the Highest Location

the calculation formula is as shown in

\begin{equation*} \sigma =\sqrt {\frac {1}{N}\left ({\sum \nolimits _{i=1}^{N}\left ({v_{i}-\bar v}\right)^{2}}\right)}\tag{4}\end{equation*}

where $\sigma $ is the V-variation; $V_{i}$ is the instantaneous speed; $\bar v$ is the average speed; The V-variation reflects the degree of speed dispersion.

#### 14) Maximum Speed by the Affected Arm After the Ball Arrived at the Highest Location

Observe the patient’s maximum movement ability. The calculation formula is the same as in (2).

#### 15) Average Speed of the Affected Arm After the Ball Arrives at the Highest Location

Observe the patient’s movement variance trend. The calculation formula is the same as in (3).

#### 16) V-Variation by Affected Arm After the Ball Arrives at the Highest Location

Observe the patient’s motor ability. If the V-variation is higher after the ball arrives at the highest location, then the patient has better motor ability. The calculation formula is the same as in (4).

#### 17) Maximum Horizontal Extension Distance of Arm Movement

Observe the maximum horizontal range of the patient’s arm movement.

#### 18) Maximum Vertical Extension Distance of Arm Movement

Observe the maximum vertical range of the patient’s arm movement.

#### 19) Maximum Extension Distance From Arm Movement in the Front-View Direction

Observe the maximum range of patient’s arm movement.

### C. Clinical Trials

#### 1) Participants

Twenty subjects were enrolled in the experiment. The demographic information of participants is shown in Table 1.

**TABLE 1:** Demographic

Gender	Number	Average Age (SD)
Male	12	68.25 (4.12)
Female	8	71.63 (5.34)

The enrollment criteria were:

a) *It was* $a$ *unilateral stroke.*
b) *The stroke took place within the last year.*
c) *The stroke diagnosis was based on an MRI, Computed Tomography, and neurological examination.*
d) *The proximal movement of the upper stroke limb reached the Browns fourth stage (inclusive) with dyskinesia*.
e) *No obvious cognitive deficit (short intelligence test score* $\le $ *20 points).*
f) *Capable of following simple instructions and understanding the experimental process and rehabilitation.*
g) *Willing to join and sign the consent form. The experimental site was at the rehabilitation department of Taipei Veterans General Hospital.*

#### 2) Procedures

Participants were informed of the experimental procedure and the purpose of the research. Participants were required to sign a consent form. In addition, participants were required not to participate in other rehabilitation training activities.

The participants attended 60 min stroke-rehabilitation sessions three times per week for eight weeks, and all participants completed the 24 training sessions. To avoid extreme fatigue from continuous training, at least one day off was provided between each session. Therapists could adjust the degree of difficulty according to the participants’ evaluation. The degree of difficulty of each training was set to satisfy the motor ability limit of the participant. Clinical assessments were conducted three days before training (0 week), within three days after training (eight-week endpoint).

#### 3) Measurement

Traditional evaluation scales such as FMA, TEMPA, and WMFT have strict assessment methods, procedures and standards. If a participant scores higher on the scale, then the participant has a better recovery status. If a participant’s score lower on the scale, then the participant has a worse recovery status. Because our research focuses on the upper limbs, not all items on the traditional evaluation scale were used. The FMA score range is 0 to 22 points, the TEMPA score range is 0 to 27 points, and the WMFT score range is 0 to 5 points.

#### 4) Analysis Method

Because the number of samples was small, data distribution was unclear and the evaluation values of the same patient’s pretest and posttest data were related variables, we employed the Wilcoxon rank-4sum test, a nonparametric test used to analyze whether the pretest and posttest data were significantly different, thereby testing the effectiveness of the bi-lateral VR task. We also used Spearman correlation analysis and a double-tailed test to evaluate the correlation between the motor indicators and the traditional evaluation scales [41].

### D. Development of Evidence-Based Assessment Model

**FIGURE 3.**
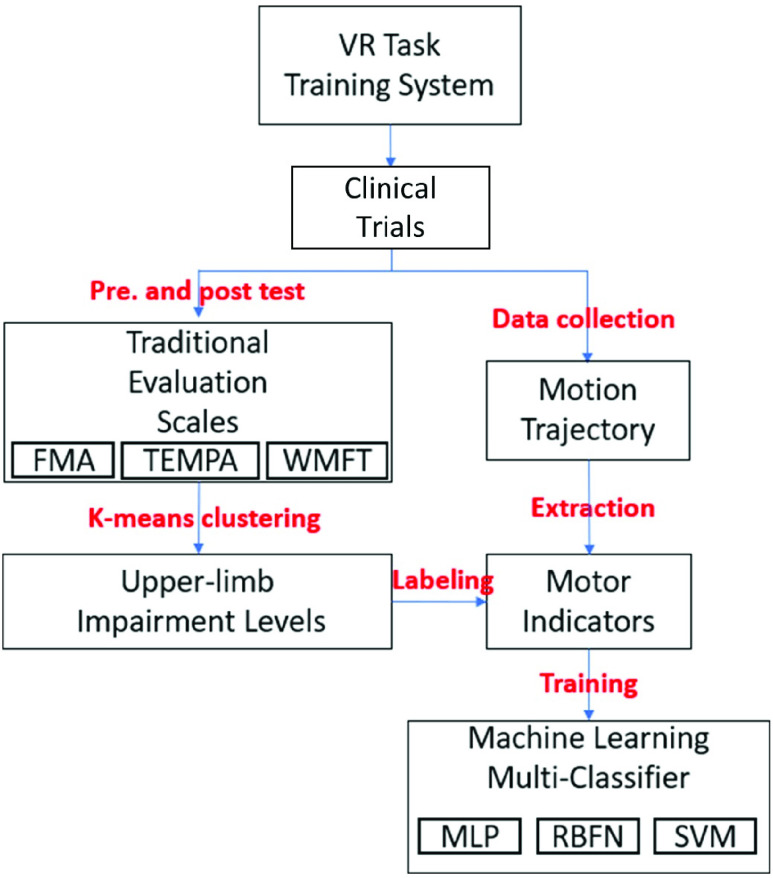
Flow diagram of the assessment model for our VR stroke-rehabilitation system. First, K-means clustering based on evaluation scales was used to cluster participants’ data. Next, the skeleton data were collected when a participant was performing the VR task via Kinect, and the features were extracted as motor indicators. Finally, multi-classifiers (MLP, RBFNN, SVM) based on evaluation scales were adopted to develop an evidence-based assessment model.

Based on development of an evidence-based, convincible assessment model for stroke rehabilitation, we followed the flow diagram in Fig. 3. First, we performed clustering (K-means) based on evaluation scales to cluster participants’ data. Next, we collected skeleton data which were collected when a participant was performing the VR task via Kinect and extracted features by motor indicators. Finally, we fit multi-classifiers (MLP, RBFNN, SVM) based on evaluation scales, thereby developing an evidence-based assessment model.

#### 1) Clustering of Multi-Dimensional Evaluation Scales

Clustering of multi-dimensional evaluation scales Traditional evaluation scales are reliable, convincible and highly recognized by therapists. To further evaluate the patients’ recovery status, we used clustering analysis based on traditional evaluation scales’ scores to place each patient into the three impairment levels. Each level could be seen as one patient’s recovery status.

The K-means algorithm was used in clustering analysis. The K-means algorithm repeatedly minimized the squared error between the empirical mean of a cluster and the individual data points [37]. Traditional evaluation scale results of 20 participants were mapped into three dimensional coordinates (x; y; z) in the form of (FMA, TEMPA, WMFT). Different K values were taken to classify the data with the K-means algorithm. The clustering effect of each K value was validated by the silhouette coefficient [42], thereby determining the appropriate K value.

#### 2) Assessment Model by Machine Learning

Based on the K-means clustering algorithm with traditional evaluation scales’ scores, we classified the participants’ evaluation scales’ scores into different clusters. Therefore, we were able to establish a classification model based on these labels. We used a machine learning-based classification algorithm to build an assessment model that could automatically classify stroke patients into different impairment levels.

The machine learning-based classification algorithms used in this study are composed of neural networks and support vector machines. Neural network is an artificial neural network formed by the interconnection of neurons for solving artificial intelligence problems. Neural networks have many different architectures. In this paper, a MLP (Multilayer Perceptron) and a RBFN (Radial Basis Function Network) were used. A Support Vector Machine (SVM) is a supervised machine learning method. It is widely used when there is a small number of samples and nonlinear and high-dimensional pattern recognition is needed. However, SVM might be incapable of multivariate data. In this case, LibSVM is often used to analyze multivariate experimental data [43].

To validate the effectiveness of the assessment model, we used the impairment level classified by K-means with traditional evaluation scales as the expected value. Some of the motor indicators chosen as input features for the assessment model have been validated for correlation between motor indicators and traditional evaluation scales. To evaluate the assessment model, we tested the accuracy and the mean absolute percentage error (MAPE) for MLP, RBFN and SVM classifiers. The MAPE’s calculation formula is shown in equation (5).

\begin{equation*} MAPE=\frac {1}{T} \sum _{k=1}^{T}\left |{\frac {d_{k}-y_{k}}{d_{k}}}\right | \times 100 \%\tag{5}\end{equation*}

where, $d_{k}$ is the actual value of the $k^{th}$ data; $y_{k}$ is the predicted output value of the $k^{th}$ data; $T$ is the total amount of data. The relationship between MAPE and prediction effect is defined as when MAPE < 10, the prediction effect is accurate; when $50\le $ MAPE, the prediction effect is inaccurate.

## IV. Results

### A. Comparison of Pretest and Posttest Data in Traditional Evaluation Scales

The comparison of pretest and posttest data in three traditional evaluation scales is shown in Table 2. The scores of all evaluation scales were significantly improved (P < 0:05), indicating the bi-lateral VR task has significant efficacy for the rehabilitation of stroke patients.

**TABLE 2:** Traditional Evaluation Scale Statistical Results

Scales	Pretest	Posttest	P-value
FMA	14.60 (4.39)	17.15 (3.60)	$0.001\ast \ast $
TEMPA	−12.60 (7.93)	−10.35 (6.63)	$0.005\ast \ast $
WMFT	3.66 (0.77)	3.84 (0.67)	$0.020\ast $

Significance Level = 0.05; $\ast \text{P} < 0.05$; $\ast \ast $ P < 0.01.

### B. Correlation Analysis of Motor Indicators and Traditional Evaluation Scales

**FIGURE 4.**
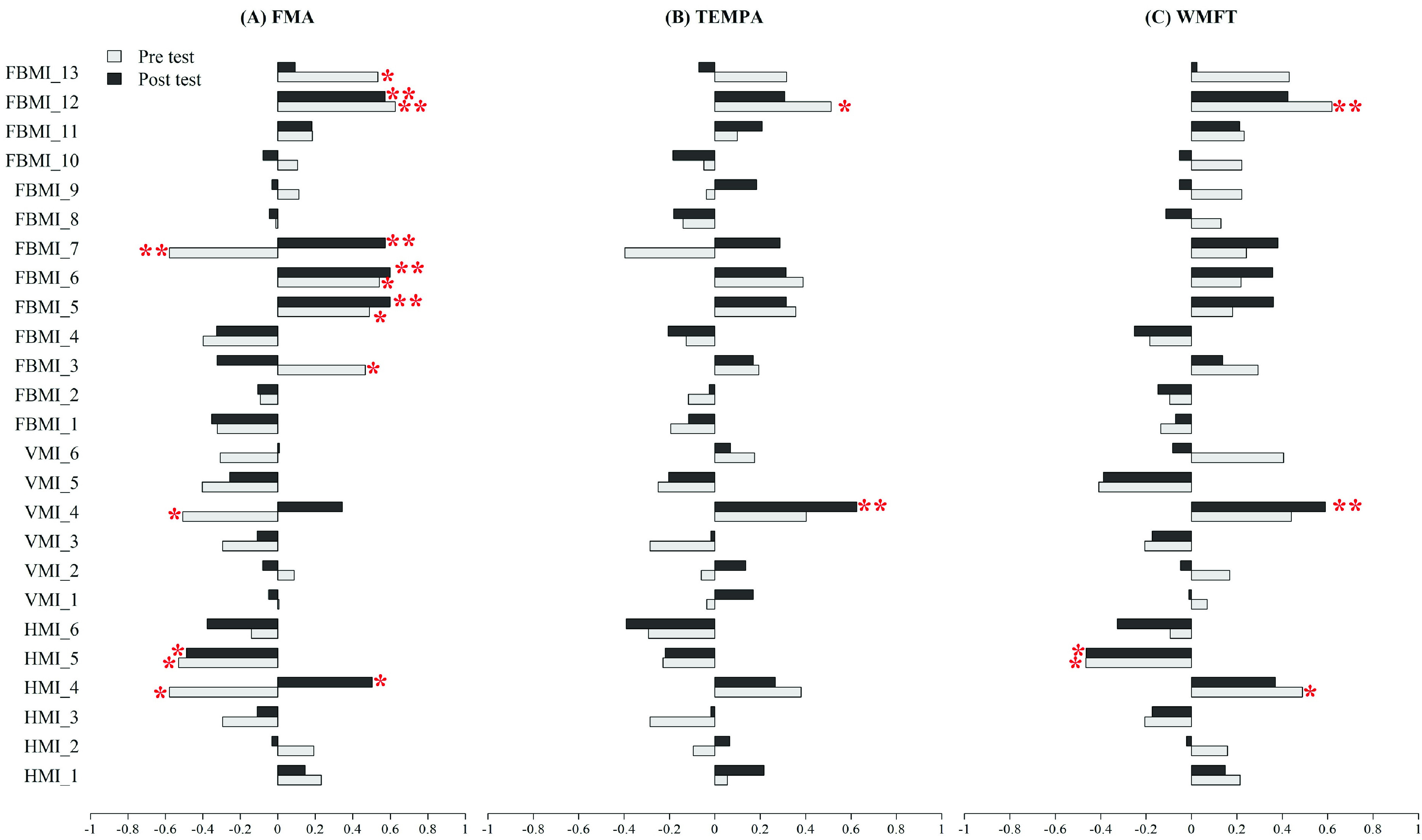
Correlation coefficients between motor indicators including horizontal motor indicator (HMI), vertical motor indicator (VMI), and full body’s motor indicator (FBMI), and traditional valuations scales which were FMA, TEMPA, and WMFT in Pretest and Posttest, respectively. Note that * means P < 0.05 and ** means P < 0.01. H(V)MI_1 = first time entering the catching range evaluated by horizontal(vertical) motor indicator; H(V)MI_2 = last time entering the catching range evaluated by horizontal(vertical) motor indicator; H(V)MI_3 = catching time evaluated by horizontal(vertical) motor indicator; H(V)MI_4 = aiming time evaluated by horizontal(vertical) motor indicator; H(V)MI_5 = amount of time between stabilization and catching ball evaluated by horizontal(vertical) motor indicator; H(V)MI_6 = number of times getting in and out of catching range evaluated by horizontal(vertical) motor indicator; FBMI_1 = catching ball time difference between normal arm and affected arm; FBMI_2 = catching ball time cost by affected arm; FBMI_3 = movement distance by affected arm; FBMI_4 = movement difference between affected arm and normal arm; FBMI_5 = maximum speed by affected arm before ball arrives at the highest location; FBMI_6 = average speed by affected arm before ball arrives at the highest location; FBMI_7 = V -variation by affected arm before ball arrives at the highest location; FBMI_8 = maximum speed by affected arm after ball arrives at the highest location; FBMI_9 = average speed by affected arm after ball arrives at the highest location; FBMI_10 = V -variation by affected arm after ball arrives at highest location; FBMI_11 = maximum extension distance from arm movement in the horizontal direction; FBMI_12 = Maximum extension distance from arm movement in the vertical direction; FBMI_13 = maximum extension distance from arm movement in the front-view direction.

The correlation between horizontal motor indicators, vertical motor indicators and traditional evaluation scales in pretest and posttest data are shown in Fig. 4 (A) and Fig. 4 (B). Aiming time and the amount of time between stabilization and catching correlate significantly with FMA, TEMPA, and WMFT in pretest and posttest. However, the results were not as expected. Aiming time showed positive correlation but the amount of time between stabilization and catching showed negative correlation. We expected that if aiming time was shorter, the amount of time between stabilization and catching would be longer if participants had better motor ability. This result might be due to the degree of difficulty setting for the bi-lateral VR task.

The correlation between full body motor indicators and traditional evaluation scales in pretest and posttest data are shown in Fig. 4 (C). Maximum speed, average speed, *V-variation* by the affected arm before the ball arrives at the highest location and maximum extension distance from arm movement in the vertical direction correlates significantly with FMA, TEMPA, and WMFT in pretest and posttest data. Each of these motor indicators are related to muscle control ability. In addition, we expected that a faster upper-limb movement speed would mean the participant has better motor ability. However, according to Fig. 4 (C), many motor indicators have no correlation with traditional evaluation scales in pretest and posttest data. This result might be due to the small number of samples and need further investigation.

### C. Cluster Analysis Based on Multi-Dimensional Evaluation Scales

**FIGURE 5.**
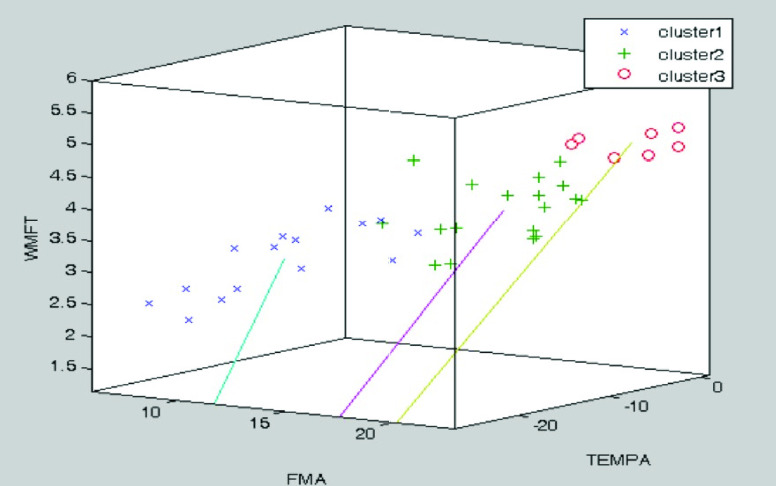
3D scatter plot for the traditional evaluation scales, Fugl-Meyer Assessment (FMA) [6], Wolf Motor Function Test (WMFT) [7], and Test D’évaluation Des Membres Supérieurs Des Personnes Âgées (TEMPA) [8]. K-means clustering was based on them, and the highest silhouette value was attained when K = 3. The blue cross, green plus, and red circle are Cluster 1, Cluster 2, and Cluster 3, respectively. The corresponding number of data are 15, 18, and 7 respectively.

The clustering analysis based on the K-means algorithm is used to determine the most proper K value. By fitting the clustering model by the silhouette coefficient, we got the highest silhouette value when K = 3. Therefore, the patient’s impairment level was divided into three categories based on the multidimensional evaluation scales, with a total of 40 points (20 patients’ evaluation scale data in pretest and posttest data). The spatial clustering result when K = 3 is shown in Fig. 5. As shown, the number of data in Cluster1, Cluster2, and Cluster3 are 15, 18, and 7 respectively.

**FIGURE 6.**
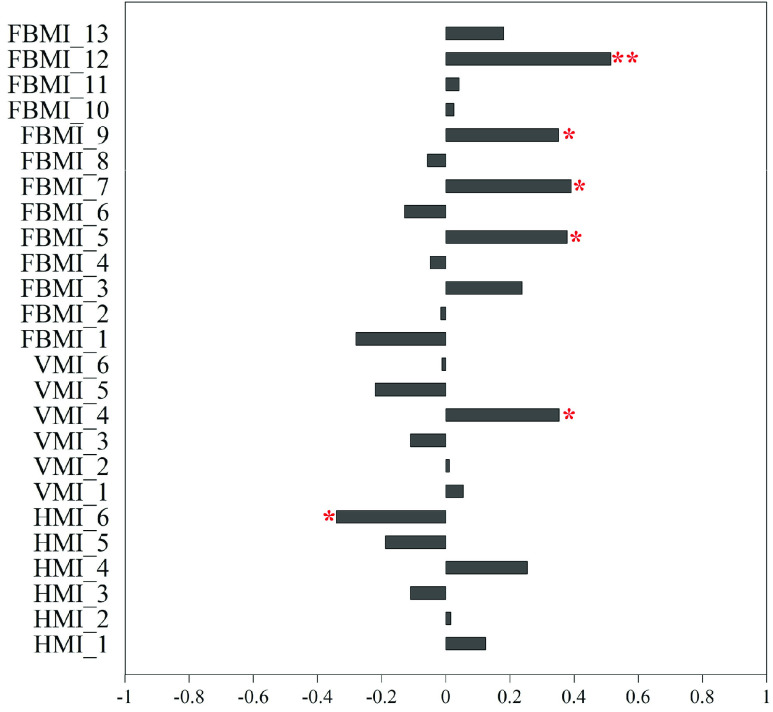
Correlation coefficients between motor indicators including horizontal motor indicator (HMI), vertical motor indicator (VMI), and full body’s motor indicator (FBMI), and corresponding cluster. Note that * means P < 0.05 and ** means P < 0.01.

Based on Fig. 4, traditional evaluation scales were replaced with the clustering result in reanalyzing the correlation between the motor indicators and the clustering result, as shown in Fig. 6. A total of six motor indicators correlates with the clustering results. These 6 indicators are the number of times getting in and out of catching range, aiming time, maximum speed, *V-variation* by affected arm before ball arrives at the highest location, average speed by affected arm after the ball arrives at the highest location, and maximum extension distance from arm movement in the vertical direction.

### D. Machine Learning Based Classification Model

Based on the above correlation analysis, we found that some motor indicators correlate with traditional evaluation scales. These motor indicators were used to train MLP, RBFN, and SVM classifiers. The initial hyperparameters, shown in Table 3, are aimed to fit the classifiers based on the overall MAPE. In neural network analysis, we first chose all (19) motor indicators to the fit neural network, as shown in Table 4. The optimal number of neurons in MLP is two, and the number of optimal neurons in RBFN is three. We also chose subclasses of motor indicators to fit MLP, RBFN classifiers, as shown in Table 5. In SVM analysis, we also chose all motor indicators and subclasses of motor indicators to fit SVM classifier. The classification result of SVM is shown in Table 6. Based on above the classification result, which uses a two-neuron architecture with all motor indicators, has the best performance of about 86% overall accuracy and 12.33% MAPE. In contrast, the highest accuracy of SVM is only 61.5%.

**TABLE 3:** Hyperparameter Settings of Machine Learning Models

Models	Parameters	Motor indicators			
		All (25)	Pretest - posttest (7)	Posttest - pretest (7)	Clustering (6)
MLP	Learning efficiency	0.5	0.5	0.5	0.5
	MSE	0.007	0.02	0.012	0.03
	Learning times	80,000	80,000	80,000	80,000
RBFN	Learning efficiency	0.5	0.5	0.5	0.5
	MSE	0.07	0.08	0.06	0.04
	Learning times	80,000	80,000	80,000	80,000
RBFN	cost	512	32	2	32
	gamma	0.08	0.5	0.5	0.008

**TABLE 4:** Classification Results of Neural Network and All Motor Indicators

	Number of neurons								
	2			3			4		
Models	Training Accuracy	Test Accuracy	Overall Accuracy	Training Accuracy	Test Accuracy	Overall Accuracy	Training Accuracy	Test Accuracy	Overall Accuracy
	Training MAPE	Test MAPE	Overall MAPE	Training MAPE	Test MAPE	Overall MAPE	Training MAPE	Test MAPE	Overall MAPE
MLP	89.38%	72.5%	86%	86.25%	67.5%	82.5%	86.25%	67.5%	82.5%
	10.33%	20.31%	12.33%	11.34%	22.07%	13.49%	11.07%	22.71%	13.4%
RBFN	81.25%	60%	77%	87.5%	63%	82.6%	84.38%	63%	80.1%
	13.86%	29.24%	16.94%	11.06%	30.67%	14.98%	12.68%	30.73%	16.29%

**TABLE 5:** Classification Results of Neural Networks and Selected Motor Indicators

	MLP (2 neurons)			RBFN (3 neurons)		
Motor indicator	Training Accuracy	Test Accuracy	Overall Accuracy	Training Accuracy	Test Accuracy	Overall Accuracy
	Training MAPE	Test MAPE	Overall MAPE	Training MAPE	Test MAPE	Overall MAPE
Pretest - posttest (7)	83.13%	60%	78.5%	81.25%	60%	77%
	16.68%	24.06%	18.1%	14.78%	35.49%	18.92%
Posttest - pretest (7)	83.13%	52.5%	77%	84.38%	60%	79.5%
	11.18%	28.84%	14.71%	8.09%	33.66%	13.2%
Clustering (6)	78.75%	60%	75%	78.12%	55%	73.5%
	19.94%	27.29%	21.41%	17.11%	35.98%	20.88%

**TABLE 6:** Classification Results of SVM and Motor Indicators

Motor indicator	Training Accuracy	Test Accuracy	Overall Accuracy
All (25)	51.88%	45%	50.5%
Pretest - posttest (7)	56.25%	42.5%	53.5%
Posttest - pretest (7)	51.88%	55%	52.5%
Clustering (6)	61.88%	60%	61.5%

## V. Discussion

According to Table 1, bi-lateral VR task could be effectively used as rehabilitation training for stroke patients. However, based on the clustering analysis above, some motor indicators unexpectedly have less correlation with traditional evaluation scales.

According to Fig. 4 some of the motor indicators have significant correlation with traditional evaluation scales in pretest and posttest as expected. For example, maximum speed, average, V -variation by affected arm before the ball arrives at the highest location are all positive correlations with traditional evaluation scales. However, some of the motor indicators unexpectedly have less correlation with traditional evaluation scales in pretest and posttest data. We believe that if the number of samples increases in the future, these motor indicators would have significant correlation with all traditional evaluation scales.

For the assessment model’s selection of motor indicators, we analyzed the correlation between horizontal, vertical, full body motor indicators and clustering in Fig. 6. We discovered that aiming time, maximum speed, V -variation by affected arm before the ball arrives at the highest location, maximum extension distance from arm movement in the vertical direction have both significant correlation with traditional evaluation scales and clustering. These motor indicators might be more important to the assessment model.

The number of neurons was considered in constructing a neural network classifier with all motor indicators. The optimal MLP and RBFN neural network models with all motor indicators were developed, as shown in Table 4. In considering the selection of motor indicators, we tried different subclasses of motor indicators to fit MLP and RBFN neural network classifiers, as shown in Table 5. However, the classification model with the highest overall accuracy and highest overall MAPE was trained by all motor indicators. We considered that although some motor indicators might not have significant correlation with traditional evaluation scales in a traditional statistics aspect, these motor indicators still impact the neural network-based classification model. The neural network models could learn implicit relations between those motor indicators. Moreover, the number of motor indicators that correlate to traditional evaluation scales might be too small for neural network models to learn well. Therefore, our neural network model still shows great potential.

According to Table 6, the SVM classification model trained with motor indicators considered traditional evaluation scales. The motor indicators with significant correlation to clustering show the highest overall accuracy in the SVM classification model. However, results of overall accuracy in Table 6 are not great.

Although several studies have devoted on developing VR system for stroke rehabilitation, it is still lack of a system which incorporated both sensor and traditional evaluation scales. More specifically, previous research adopted VR training tasks in stroke rehabilitation to show the efficiency of VR rehabilitation by comparing pretest and posttest data without sensor data and traditional evaluation scales [31], [32], [33], [34]. While several studies only considered the sensor data to implement the evaluation [25], [26], [27], [28], [35], [37], [38], [39]. Our system was developed by considering both sensor data and traditional evaluation scales. Since the VR systems lack of similarity in previous research, comparing the results such as the performance of the proposed models would have no meaning. Yet, our results have shown that considering both sensor data and traditional evaluation scales is helpful to develop the VR system.

Because the number of samples is small, the results might not necessarily be accurate. Nonetheless, an automated motor assessment model is feasible. If we have enough samples in the future, we would consider doing the following: (1) Increase the type of evaluation scales. (2) Increase the number of clusters. (3) Implement additional motor indicators.

## VI. Conclusion

In this paper, we developed a VR-based stroke rehabilitation system for the upper limbs. We proposed a motor assessment model with 20 stroke participants’ traditional evaluation scales (FMA, TEMPA and WMFT). First, we used the K-means algorithm to cluster participants’ pretest and posttest data. Furthermore, we designed many motor indicators for a bilateral VR task and analyzed their correlation with traditional evaluation scales. Maximum speed, average speed, V-variation by the affected arm before the ball arrives at the highest location and maximum extension distance from arm movement in the vertical direction correlates significantly with FMA, TEMPA, and WMFT in pretest and posttest data. Each of these motor indicators are related to muscle control ability. Moreover, we used clustering analysis and the correlation between motor indicators and traditional evaluation scales to fit the machine learning-based classification model (MLP, RBFN, SVM). Finally, our motor assessment model with MLP architecture achieved 86% overall accuracy. In the future, we would perform a larger clinical trial to enhance the accuracy of our assessment model. We would also adopt advanced machine learning methods such as DNN, RNN, and LSTM to exploit our Kinect-based skeleton data to implement a higher performance motor assessment model.
